# Acute Forearm Compartment Syndrome Following Physical Therapy in a Patient Receiving Anticoagulation Therapy: A Case Report

**DOI:** 10.3390/jcm15135052

**Published:** 2026-06-29

**Authors:** Dong Wan Kim, Heui Ro Na, Seung Hyun Kim, Jun Ho Choi, Jae Ha Hwang, Kwang Seog Kim

**Affiliations:** Department of Plastic and Reconstructive Surgery, Chonnam National University Hospital, Chonnam National University Medical School, Gwangju 61469, Republic of Korea; waaan37@gmail.com (D.W.K.); skgmlfh1@naver.com (H.R.N.); hi_1004@naver.com (S.H.K.); cjh_0502@hanmail.net (J.H.C.); pskim@chonnam.ac.kr (K.S.K.)

**Keywords:** compartment syndromes, forearm injuries, anticoagulants, warfarin, physical therapy

## Abstract

**Background**: Acute compartment syndrome is a surgical emergency that is most often associated with trauma. Rarely, however, it can develop without major trauma in patients receiving anticoagulation therapy. **Methods**: A 53-year-old woman receiving warfarin therapy presented with progressive swelling and pain in the left forearm after physical therapy. Computed tomography angiography showed preserved arterial flow. On clinical examination, pain and paralysis were present, whereas pallor and paresthesia were absent. Direct compartment pressure measurements demonstrated markedly elevated pressures in the dorsal and deep volar compartments, measuring 88 mmHg and 110 mmHg, respectively. Emergency fasciotomy was performed approximately 9 h after symptom onset. **Results**: Intraoperative findings showed hematoma formation and partial muscle necrosis in the deep volar compartment. After surgery, persistent bleeding required repeated hemostatic interventions and blood-product transfusion. Negative-pressure wound therapy was applied, and delayed primary closure was subsequently performed. Anticoagulation therapy was temporarily discontinued and later resumed. The patient recovered without further bleeding, wound complications, or functional impairment and was discharged in stable condition. No complications, including hematoma recurrence or infection, were observed during 6 months of follow-up. **Conclusions**: This case highlights a temporal association between physical therapy and acute forearm compartment syndrome in a patient receiving anticoagulation therapy. It also emphasizes that preserved peripheral pulses and intact arterial flow do not exclude the diagnosis. Early recognition and prompt surgical intervention remain essential to prevent irreversible tissue damage and optimize outcomes.

## 1. Introduction

Acute compartment syndrome develops through a progressive cycle of increased intracompartmental pressure, impaired microvascular perfusion, tissue ischemia, and edema formation. As tissue perfusion decreases, capillary permeability increases, resulting in further fluid accumulation within the confined compartment. Without timely intervention, this vicious cycle may ultimately lead to muscle necrosis, nerve injury, contracture, and permanent functional impairment. Because irreversible tissue damage can occur within several hours, compartment syndrome is considered a true surgical emergency requiring prompt diagnosis and decompression [1,2,3].

Although most cases are associated with fractures or high-energy trauma, non-traumatic causes have also been reported [1,3]. Patients receiving anticoagulation therapy are particularly vulnerable to spontaneous bleeding or bleeding after minor trauma, either of which may result in compartment syndrome [4,5]. Physical therapy is generally regarded as a safe and beneficial intervention and is commonly prescribed for patients with neurologic or musculoskeletal disorders. However, patients receiving anticoagulation therapy may be more susceptible to hemorrhagic complications following minor tissue injury or mechanical stress [4,5,6].

We report a rare case of acute forearm compartment syndrome that developed following physical therapy in a patient receiving anticoagulation therapy.

## 2. Case Presentation

A 53-year-old woman presented to the emergency department with progressive swelling and pain in the left forearm after undergoing physical therapy. Her symptoms worsened over several hours, and she visited the emergency department approximately 7 h after symptom onset. She had a history of atrial fibrillation and was receiving warfarin anticoagulation therapy. Laboratory evaluation on admission demonstrated a prolonged coagulation profile consistent with anticoagulation therapy, including a prothrombin time (PT) of 27.3 s and an international normalized ratio (INR) of 2.57. Activated partial thromboplastin time (aPTT) was mildly prolonged at 40.8 s. Hemoglobin and platelet count were 14.8 g/dL and 195 × 10^9^/L, respectively. D-dimer and fibrin degradation product levels were not elevated. Her other medications included atorvastatin and digoxin. She had experienced a right middle cerebral artery infarction in 2010, for which she received thrombolysis and anticoagulation therapy. As a sequela, residual flexor contracture of the left wrist persisted, and she had been undergoing periodic physical therapy. Her medical history was also notable for mitral stenosis, mitral regurgitation, aortic insufficiency, and prior mitral balloon valvuloplasty.

Detailed information regarding the specific physical therapy modality was unavailable because the treatment had been performed at an outside institution. According to the patient, the session primarily involved stretching and mobilization exercises of the affected upper extremity.

Physical examination revealed pain and paralysis, whereas pallor and paresthesia were absent. Peripheral pulses were preserved. Computed tomography angiography demonstrated intact arterial flow (Figure 1). However, direct compartment pressure measurements were markedly elevated, with pressures of 88 mmHg in the dorsal compartment and 110 mmHg in the deep volar compartment. Based on these findings, acute compartment syndrome of the left forearm was diagnosed.

Emergency fasciotomy was performed approximately 9 h after symptom onset. Intraoperatively, a hematoma was identified in the proximal forearm. Partial muscle necrosis was present in the deep volar compartment, whereas the other compartments remained viable. Hematoma evacuation and decompression were performed (Figure 2). The wound was left open after fasciotomy without primary closure and was managed with regular dressing changes until stabilization permitted delayed closure. Postoperative laboratory studies demonstrated a progressive decrease in hemoglobin level from 14.8 g/dL at presentation to 13.6 g/dL and subsequently 11.5 g/dL during the early postoperative period. Coagulation studies remained prolonged, with a PT of 19.5 s and INR of 1.80. Persistent postoperative bleeding was subsequently noted. Hemostasis required repeated interventions, including epinephrine-soaked gauze packing, bipolar electrocoagulation, topical hemostatic agents, and transfusion of fresh frozen plasma and packed red blood cells. Negative-pressure wound therapy was applied, and serial wound assessments showed a gradual reduction in bleeding without evidence of infection. Delayed primary closure was performed 5 days after fasciotomy, once the wound had stabilized (Figure 3). Anticoagulation therapy was temporarily withheld and subsequently resumed with heparin bridging followed by warfarin. The patient recovered without further bleeding or wound complications and was discharged in good condition. She was advised to exercise caution during future physical therapy to reduce the risk of recurrent hematoma.

The patient was followed regularly in the outpatient clinic for 6 months (Figure 4). At the 6-month follow-up, the patient demonstrated full return to her preinjury functional status without recurrent swelling, neurologic symptoms, or clinically significant restriction of wrist and finger motion.

## 3. Discussion

Acute compartment syndrome is most commonly associated with trauma [1,3]; however, non-traumatic etiologies, including anticoagulation-related bleeding, have been increasingly recognized [4,5]. In the present case, compartment syndrome developed after physical therapy despite the absence of a significant traumatic event. This clinical course suggests that clinically significant bleeding may occur following seemingly minor mechanical stress in patients receiving anticoagulation therapy [6].

Although acute compartment syndrome is classically associated with fractures and high-energy trauma, atraumatic cases account for a small but clinically important proportion of presentations. Among these, anticoagulation-associated hemorrhage has emerged as a recognized cause, particularly in elderly patients and those receiving long-term anticoagulant therapy [4,5]. Previous reports have described compartment syndrome developing after spontaneous bleeding or seemingly minor injuries in anticoagulated patients, emphasizing that the severity of the precipitating event may not correlate with the severity of the resulting compartment syndrome [5,6]. Consequently, clinicians should maintain a high level of suspicion when evaluating anticoagulated patients who present with progressive pain and swelling, even in the absence of significant trauma. The characteristics of previously reported anticoagulation-associated upper-extremity compartment syndrome cases and the present case are summarized in Table 1.

The pathophysiology of compartment syndrome is characterized by a progressive cycle of increased intracompartmental pressure, impaired microvascular perfusion, tissue ischemia, and edema formation [2,3]. As tissue perfusion decreases, capillary permeability increases, resulting in further fluid accumulation within the confined compartment. Without timely intervention, this vicious cycle may ultimately lead to muscle necrosis, nerve injury, contracture, and permanent functional impairment. In the present case, expansion of an intramuscular hematoma associated with anticoagulation therapy likely initiated this pathophysiologic cascade.

Warfarin therapy likely contributed substantially to both the development of compartment syndrome and the complexity of the postoperative course. The patient experienced persistent postoperative bleeding that required multiple hemostatic interventions and transfusions. This finding underscores the need to consider bleeding tendency not only as a possible cause of compartment syndrome but also as an important factor in postoperative management, particularly in patients receiving anticoagulation therapy, in whom effective hemostasis may be difficult to achieve even during minor procedures [7]. In addition, staged wound management, including negative-pressure wound therapy and delayed closure, was required because of ongoing bleeding and soft-tissue swelling [8]. Anticoagulation management was also challenging, requiring temporary discontinuation followed by cautious reinitiation to balance the risks of bleeding and thromboembolism.

Physical therapy is generally regarded as a safe and beneficial intervention for patients with neuromuscular dysfunction. In the present case, symptoms developed shortly after a physiotherapy session; however, the exact mechanism linking the intervention to the subsequent hematoma formation remains unclear. Although a temporal association was observed, a direct causal relationship between the physiotherapy intervention and the development of compartment syndrome cannot be established based on a single case report. In addition to surgical evacuation, alternative approaches to hematoma management have been reported. Hyaluronidase facilitates the breakdown of hyaluronic acid in the extracellular matrix and thereby enhances the absorption of hematoma and edema by increasing tissue permeability and diffusion [9]. A clinical study has shown that hyaluronidase injection can accelerate hematoma resolution and reduce fibrosis formation after trauma [10]. Although hyaluronidase was not used in the present case because acute surgical decompression was indicated, its potential relevance lies in the management of residual or localized hematomas, particularly in patients at increased risk of bleeding complications. Therefore, while it cannot replace fasciotomy in established compartment syndrome, hyaluronidase may be considered as an adjunctive treatment in selected cases involving less severe hematoma or during the subacute phase.

Another important feature of this case is that compartment syndrome occurred despite preserved peripheral pulses and intact arterial flow on imaging [1]. These findings can delay diagnosis if clinicians rely solely on vascular status. The diagnosis of acute compartment syndrome remains primarily clinical and is based on the presence of severe pain, pain disproportionate to the injury, pain on passive stretch, and progressive neurologic dysfunction. Although imaging studies may assist in excluding alternative diagnoses, they should not delay surgical consultation or decompression when clinical suspicion is high. In patients with progressive symptoms suggestive of compartment syndrome, timely clinical assessment remains more important than radiographic confirmation. However, these findings may be difficult to interpret in patients with altered mental status or atypical presentations.

Compartment pressure measurement serves as an important adjunctive diagnostic tool when the diagnosis is uncertain. Previous studies have suggested that either an absolute compartment pressure above 30–40 mmHg or a differential pressure (diastolic blood pressure minus compartment pressure) below 30 mmHg may indicate inadequate tissue perfusion requiring fasciotomy [2]. Nevertheless, pressure measurements should always be interpreted in conjunction with clinical findings rather than as isolated values. Serial examinations are equally important because compartment syndrome is a dynamic process and symptoms may evolve rapidly over time. In the present case, markedly elevated compartment pressures of 88 mmHg and 110 mmHg, combined with progressive symptoms, facilitated early diagnosis despite preserved arterial flow on computed tomography angiography. The relatively rapid progression of symptoms in this case further emphasizes the importance of early recognition. The interval from symptom onset to fasciotomy was approximately 9 h, and timely surgical intervention likely contributed to the favorable outcome [11,12].

Fasciotomy remains the definitive treatment for acute compartment syndrome. Once the diagnosis is established or strongly suspected, surgical decompression should not be delayed because irreversible neuromuscular injury may occur with prolonged ischemia [1]. The primary goals of fasciotomy are restoration of tissue perfusion, evacuation of hematoma when present, and prevention of further muscle and nerve injury. In the present case, markedly elevated compartment pressures, progressive symptoms, and intraoperative evidence of muscle compromise confirmed the necessity of urgent surgical intervention.

Delayed or missed diagnosis of compartment syndrome can result in devastating complications, including muscle necrosis, peripheral nerve injury, Volkmann ischemic contracture, chronic pain, infection, and permanent loss of limb function [1,2,3]. In severe cases, amputation or systemic complications such as rhabdomyolysis and acute kidney injury may occur. The absence of these complications in the present patient was likely attributable to timely diagnosis, prompt fasciotomy, and careful postoperative management.

This case is clinically noteworthy because compartment syndrome developed after a procedure generally considered safe and non-traumatic. Furthermore, diagnosis was potentially challenging because arterial flow remained intact and peripheral pulses were preserved. The case therefore highlights both the susceptibility of anticoagulated patients to significant hemorrhagic complications after minor mechanical stress and the importance of maintaining clinical suspicion even when vascular findings appear reassuring. Clinicians should maintain a high index of suspicion for compartment syndrome in patients receiving anticoagulation therapy who present with disproportionate pain after even minor events, including physical therapy.

From a rehabilitation perspective, this case highlights the importance of obtaining a thorough medication history before initiating treatment. Although physiotherapy is generally safe, clinicians should be aware that patients receiving anticoagulation therapy may be at increased risk of bleeding complications. Careful monitoring for disproportionate pain, progressive swelling, or unexpected functional deterioration is warranted, particularly when symptoms appear inconsistent with the expected response to treatment. Early recognition of these warning signs may facilitate timely referral and prevent delays in diagnosis.

## 4. Conclusions

Acute compartment syndrome may occur following seemingly minor events, including physical therapy, in patients receiving anticoagulation therapy. Clinicians should maintain awareness of the potential for bleeding complications in anticoagulated patients presenting with disproportionate pain and swelling. Early recognition and prompt fasciotomy remain essential to prevent irreversible tissue damage and achieve favorable outcomes.

## Figures and Tables

**Figure 1:**
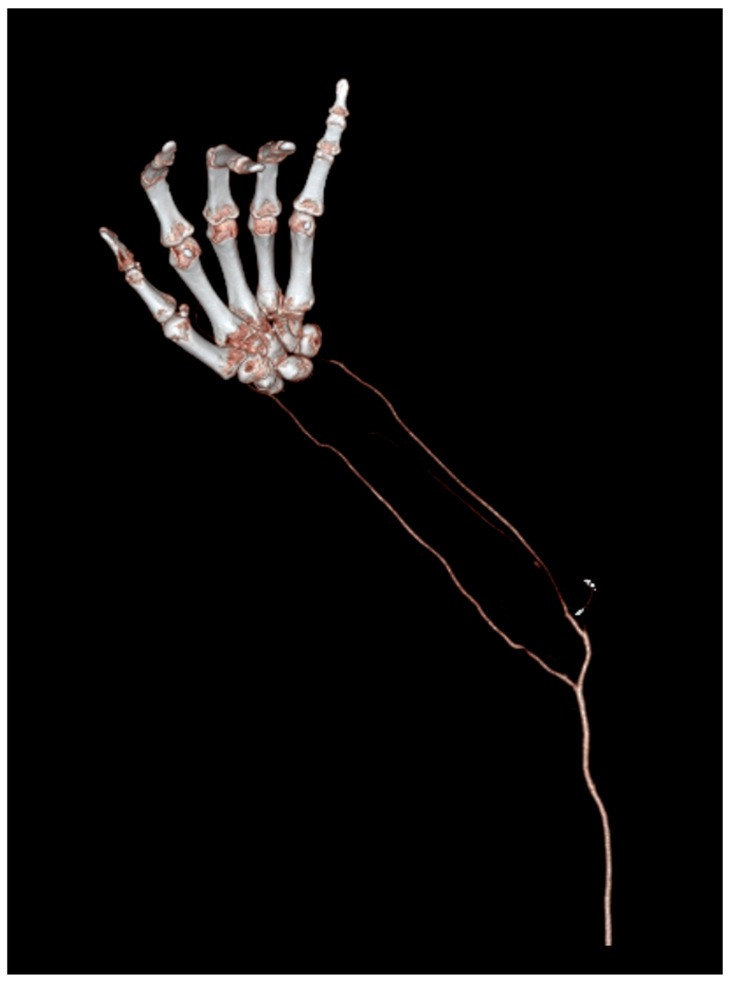
Computed tomography angiography of the left upper extremity. No definite evidence of vascular injury or active bleeding is observed, and arterial flow is preserved.

**Figure 2:**
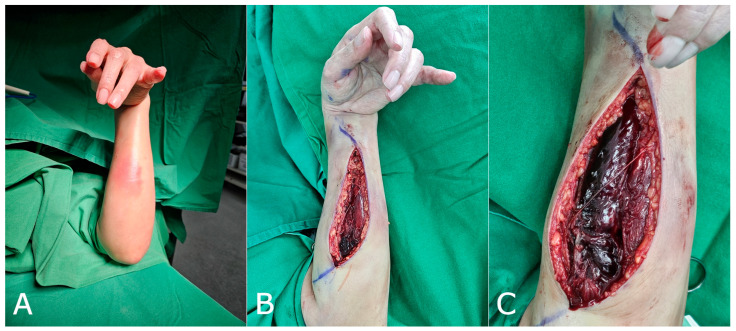
Intraoperative clinical photographs obtained at the time of emergency fasciotomy. (**A**) Preoperative appearance of the left forearm showing marked swelling and erythema suggestive of compartment syndrome. (**B**) Surgical exposure after fasciotomy. (**C**) Hematoma identified within the compartment.

**Figure 3:**
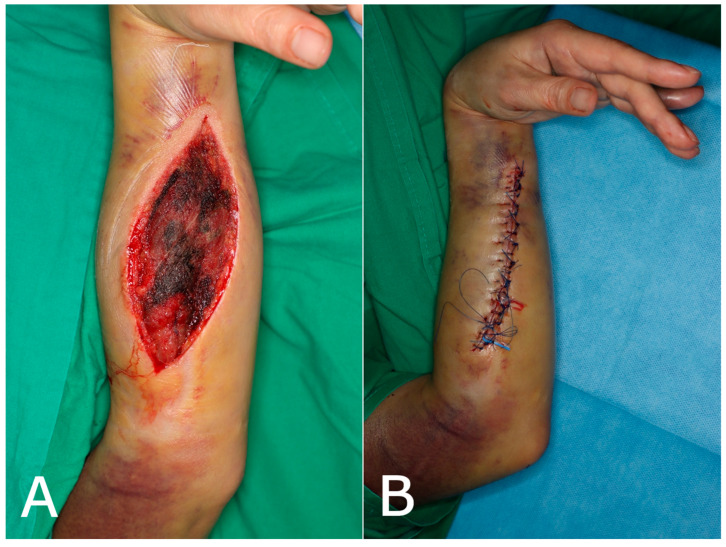
Postoperative clinical photographs. (**A**) Open wound after fasciotomy showing reduced swelling and a stabilized wound condition. (**B**) Wound after delayed primary closure performed 5 days postoperatively.

**Figure 4:**
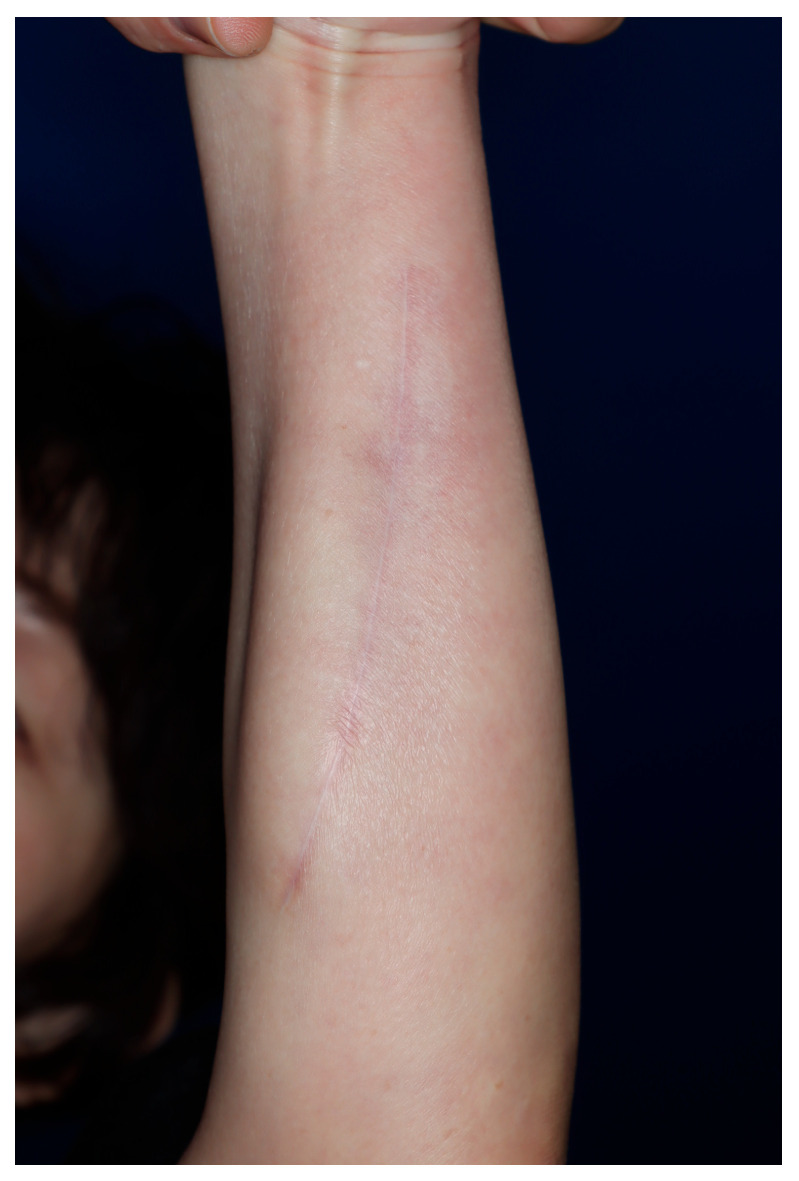
Clinical photograph obtained at the 6-month follow-up. The wound is well healed with a satisfactory cosmetic appearance and no evidence of recurrent swelling or significant scarring. The patient achieved full recovery to her preinjury functional status without neurologic symptoms or clinically significant limitation of wrist and finger motion.

**Table 1:** Published cases of anticoagulation-associated upper-extremity compartment syndrome and comparison with the present case.

Study	Age/Sex	Anticoagulant	Triggering Event	Location	Treatment	Outcome
Titolo et al. [6]	66/F	Warfarin	Minor shoulder trauma with biceps rupture	Upper arm	Fasciotomy, hematoma drainage	Near-complete recovery
Zimmerman et al. [5]	75/M	Warfarin ± antiplatelet therapy	Spontaneous hematoma	Upper arm	Fasciotomy, hematoma evacuation	Limb salvaged
Present case	53/F	Warfarin	Symptoms developed following physical therapy	Forearm	Fasciotomy, hematoma evacuation, NPWT, delayed closure	Full functional recovery at 6 months

## Data Availability

No new data were created or analyzed in this study. Data sharing is not applicable to this article.

## References

[1] Matsen F.A., Winquist R.A., Krugmire R.B. (1980). Diagnosis and management of compartmental syndromes. J. Bone Jt. Surg..

[2] Mubarak S.J., Owen C.A., Hargens A.R., Garetto L.P., Akeson W.H. (1978). Acute compartment syndromes: Diagnosis and treatment with the aid of the wick catheter. J. Bone Jt. Surg..

[3] Schmidt A.H. (2016). Acute Compartment Syndrome. Orthop. Clin. N. Am..

[4] Mazur M., Jabaly N., Ebraheim N. (2018). Acute compartment syndrome in patients on long-term anticoagulation therapy. J. Trauma Crit. Care.

[5] Zimmerman D.C., Kapoor T., Elfond M., Scott P. (2013). Spontaneous Compartment Syndrome of the Upper Arm in a Patient Receiving Anticoagulation Therapy. J. Emerg. Med..

[6] Titolo P., Milani P., Panero B., Ciclamini D., Colzani G., Artiaco S. (2014). Acute Compartment Syndrome of the Arm after Minor Trauma in a Patient with Optimal Range of Oral Anticoagulant Therapy: A Case Report. Case Rep. Orthop..

[7] Singh Jolly S., Rattan V. (2025). Is chitosan-based dressing more effective than gauze pressure in achieving early hemostasis after dental extractions in patients with deranged coagulation profiles?. Arch. Craniofacial Surg..

[8] Fonseca A.F., Herrera G.A., Gallego A., Llinas P.J. (2023). Treatment of compartment syndrome in the leg in an elderly patient with acquired hemophilia type A: A case report. Int. J. Surg. Case Rep..

[9] Amir A., Kim S., Stecco A., Jankowski M.P., Raghavan P. (2022). Hyaluronan homeostasis and its role in pain and muscle stiffness. PMR.

[10] Han J.H., Kim J., Yoon K.C., Shin H.W. (2018). Treatment of post-traumatic hematoma and fibrosis using hyaluronidase injection. Arch. Craniofacial Surg..

[11] McQueen M.M., Gaston P., Court-Brown C.M. (2000). Acute compartment syndrome: Who is at risk?. J. Bone Jt. Surg. Br..

[12] Mauffrey C., Hak D.J., Martin M.P. (2019). Compartment Syndrome.

